# High diversity and sharing of strongylid nematodes in humans and great apes co-habiting an unprotected area in Cameroon

**DOI:** 10.1371/journal.pntd.0011499

**Published:** 2023-08-25

**Authors:** Vladislav Ilík, Jakub Kreisinger, David Modrý, Erich Marquard Schwarz, Nikki Tagg, Donald Mbohli, Irène Charmance Nkombou, Klára Judita Petrželková, Barbora Pafčo

**Affiliations:** 1 Department of Botany and Zoology, Faculty of Science, Masaryk University, Brno, Czech Republic; 2 Institute of Vertebrate Biology, Czech Academy of Sciences, Brno, Czech Republic; 3 Department of Zoology, Faculty of Science, Charles University, Praha, Czech Republic; 4 Institute of Parasitology, Biology Centre, Czech Academy of Sciences, Ceske Budejovice, Czech Republic; 5 Department of Veterinary Sciences, Faculty of Agrobiology, Food and Natural Resources/CINeZ, Czech University of Life Sciences Prague, Prague, Czech Republic; 6 Department of Molecular Biology and Genetics, Cornell University, Ithaca, New York, United States of America; 7 Centre for Research and Conservation/KMDA, Antwerp, Belgium; 8 Association de la Protection des Grands Singes, Yaoundé, Cameroon; 9 University of Dschang, Dschang, Cameroon; Instituto Butantan, BRAZIL

## Abstract

Rapid increases in human populations and environmental changes of past decades have led to changes in rates of contact and spatial overlap with wildlife. Together with other historical, social and environmental processes, this has significantly contributed to pathogen transmission in both directions, especially between humans and non-human primates, whose close phylogenetic relationship facilitates cross-infections. Using high-throughput amplicon sequencing, we studied strongylid communities in sympatric western lowland gorillas, central chimpanzees and humans co-occurring in an unprotected area in the northern periphery of the Dja Faunal Reserve, Cameroon. At the genus level, we classified 65 strongylid ITS-2 amplicon sequencing variants (ASVs) in humans and great apes. Great apes exhibited higher strongylid diversity than humans. *Necator* and *Oesophagostomum* were the most prevalent genera, and we commonly observed mixed infections of more than one strongylid species. Human strongylid communities were dominated by the human hookworm *N*. *americanus*, while great apes were mainly infected with *N*. *gorillae*, *O*. *stephanostomum* and trichostrongylids. We were also able to detect rare strongylid taxa (such as *Ancylostoma* and *Ternidens*). We detected eight ASVs shared between humans and great apes (four *N*. *americanus* variants, two *N*. *gorillae* variants, one *O*. *stephanostomum* type I and one *Trichostrongylus* sp. type II variant). Our results show that knowledge of strongylid communities in primates, including humans, is still limited. Sharing the same habitat, especially outside protected areas (where access to the forest is not restricted), can enable mutual parasite exchange and can even override host phylogeny or conserved patterns. Such studies are critical for assessing the threats posed to all hosts by increasing human-wildlife spatial overlap. In this study, the term "contact" refers to physical contact, while "spatial overlap" refers to environmental contact.

## Author summary

Strongylid nematodes are common intestinal parasites, infecting a wide range of mammalian taxa, including humans and non-human primates (NHPs). They have evolved to live in vertebrate hosts for months or years and form complex communities within these hosts. Heavy strongylid infections can cause severe intestinal inflammation, intestinal lesions, respiratory difficulties, iron deficiency anemia, weight loss, childhood stunting, and adverse birth outcomes. As human population and human settlements have grown rapidly in recent decades, people often live in close proximity to wildlife, allowing for cross-transmission of soil-transmitted helminths. Because humans and NHPs (especially great apes) are closely related to one another evolutionarily, it is easy for strongylids infecting one primate species to jump to a different primate host. This evolutionary relationship can result in overlap and reciprocal exchange of pathogens and can have a damaging effect both on humans and NHP endangered species populations. As DNA-based diagnosis has become a routine part of modern parasitology, we employed a modern high-throughput sequencing approach to describe complex communities and zoonotic patterns of parasitic strongylid nematodes infecting humans and great apes in Cameroon.

## Introduction

Among parasites, strongylid nematodes are of high importance to research, because they cause one of the most common but neglected tropical diseases in humans associated with the occurrence of pathologies [1,2]. They also cause significant parasitosis in livestock, which has a major economic impact on the livestock industry worldwide. Furthermore, under certain circumstances, strongylid nematodes could be pathogenic to wildlife, including non-human primates (NHPs) [3–5]. Strongylid nematodes inhabit various parts of the host body, mainly gastrointestinal and pulmonary tract, where they feed on blood or tissues [3,6,7]. They can live for many years within their hosts and generally do not cause mortality; however, severe infections can lead to inflammatory reactions, lesions, severe weight loss, anemia or malnutrition [8] and can be attributed to cases of human as well as animal deaths [9]. In humans, the most important strongylids are hookworms (*Necator americanus*, *Ancylostoma duodenale*, and *A*. *ceylanicum*), infecting over 400 million people worldwide [10]. *Necator* hookworms and the nodule worm of the genus *Oesophagostomum* are considered the most prevalent helminths in great apes [11].

Unfortunately, identification of distinct strongylid taxa from feces using microscopy is essentially impossible, as strongylid eggs are morphologically indistinguishable [5]. Thus, strongylid identification has been mostly dependent on DNA amplification and sequence analyses [12–14]. Strongylids have mainly been genotyped through DNA amplification methods targeting only one strongylid genus, followed by Sanger sequencing. However, occurrence of complex strongylid communities makes utilization of high-throughput sequencing (HTS) essential [4,15,16]. Despite some limitations (e.g., sequencing errors, short length of the reads or increased diversity due to presence of paralogues in genomes), HTS of standard phylogenetic markers amplified from complex target populations (metabarcoding) is inexpensive and allows efficient genotyping of hundreds of samples at a time, untangling mixed infections and detecting rare taxa [17–21]. Exact delineation of amplicon sequencing variants (ASVs) can help understand the molecular epidemiology of pathogens and, consequently, HTS metagenomics has brought about a much deeper insight into the diversity of strongylid nematodes and has revealed hidden zoonotic transmissions or parasite sharing [16,22–24].

The close phylogenetic relationship between NHPs and humans significantly facilitates the overlap and transmission of pathogens and can have a damaging effect on populations of both humans and endangered NHPs [25,26]. The rapid growth of the human population and the resulting encroachment into and modification of natural animal habitats have led to an increase in physical contact and spatial overlap with wildlife, creating ideal conditions for pathogen transmission and exchange also due to changes in ecological, political, economic, and social relations [27–29]. Recently, conservation activities and tourism also contribute to transmission of human pathogens to wildlife and can threaten endangered animals [30,31]. Therefore, it is critical to monitor pathogens, including parasites, at the human-wildlife interface to detect and find ways to prevent such exchanges. Several studies have revealed the zoonotic potential of strongylid nematodes with respect to various anthropogenic disturbances; for example, *Oesophagostomum* species were found to be shared between humans and great apes in Eastern Africa [12,32] and at least two *Necator* species are shared in Central African Republic and Gabon [33,34]. Using the HTS approach, Pafčo et al. [4] observed hidden transmissions of strongylid nematodes between humans and NHPs in the forest habitats of the Central African Republic, with *Necator* spp. as a main driving force of overlap between different hosts.

We explored strongylid nematode diversity in humans and great apes cohabiting an unprotected area in the northern periphery of the Dja Faunal Reserve, Cameroon. We evaluated possible zoonotic transmission patterns and assessed the impact of behavioral/hygiene habits of the local people on their strongylid infections. We employed an ITS-2 metabarcoding approach and predicted differences in strongylid nematode communities between different primate hosts.

## Methods

### Ethics statement

The research complied with the legal requirements of the Cameroon and was approved by Ministère de la Recherche Scientifique et de l’Innovation (permit number 0000105/MINRESI/B00/C00/C10/C12) and Ministère des Forêts et de la Faune (permit number 1371/PRS/MINFOF/SG/DFAP/SDVEF/SC). The ape samples were collected noninvasively and did not affect the animals. Human sampling and data collection followed the protocol approved by the Ethics Committee of the Biological Centre of Academy of Sciences, České Budějovice, Czech Republic and was approved by the local authorities. Sampling was performed after obtaining oral and written informed consent of all registered volunteers. Samples were numbered, paired with questionnaires and anonymized.

### Study site, sample collection

Our study took place in the northern periphery of the Dja Faunal Reserve (Dja FR), located in South-East Cameroon. The reserve is part of a semi-deciduous lowland forest (500–700 m above sea level) with an equatorial and humid climate characterized by one short and one long dry season in between two rainy seasons (February–July/August–November) [35]. The unprotected area (40 km^2^), comprising the target area of Project Grands Singes (PGS), under Antwerp Zoo Society, Belgium included the research camp La Belgique and three village settlements approximately 25 km from the camp. Several ethnic groups (including the Badjoué, the Fang, the Kaka, the Nzime, the Niem and the Baka) live in the periphery of the reserve in close coexistence with wildlife [36]. Although the human population density is low, the pressure on the reserve is substantial, as crops [37], hunting [38,39] and logging [40] remain the main sources of livelihood for the local people. High densities of central chimpanzees (*Pan troglodytes troglodytes*) and western lowland gorillas (*Gorilla gorilla gorilla*) were recorded in the reserve as well as in the unprotected area around the camp La Belgique [41].

Human sampling was carried out in three villages–Duomo-Pierre, Malen V, and Mimpala (61 households and approximately 600 people in total)–and great ape samples were collected in secondary forest areas between the villages and around La Belgique research camp during September and October 2014 (major wet season peak). Fresh fecal samples (total number: *n =* 139) were collected non-invasively from humans (*n =* 48), median age 26 years, and free-ranging great apes: central chimpanzees (*n* = 31) and western lowland gorillas (*n* = 60). Human participants were provided with sampling tubes and samples were then gathered by researchers in the villages. Samples of great apes were collected from the ground under morning nests, a maximum of three hours after individuals left their nests. To reduce the risk of re-sampling of the same individuals and groups of individuals, only groups of different sizes (at the same locality) or groups of the same size (but not at the same locality) were sampled, and one sample per nest was taken. The samples were immediately fixed in 96% ethanol and stored at room temperature for a maximum of two weeks until they were sent to the Department of Pathology and Parasitology of University of Veterinary Sciences Brno, where they were stored at -20°C.

Human participants also filled out a close ended questionnaire (S1 Fig) about their lifestyle including frequency of entering the forest, interaction with great apes, clothing, hygiene, anthelmintic treatment and dietary habits (Table 1). All participants spoke French and researchers assisted them to fill in the questionnaires.

**Table 1:** Results of questionnaires based on respondents’ answers. Survey focused mainly on human-animal interactions, lifestyle and hygiene standards.

Activity	Yes	No	Frequently	Sometimes	Never	River	Well	Both
**Entering the forest**	-	-	64.6%	29.2%	6.2%	-	-	-
**Contact/Encounter with wild apes**	89.6%	10.4%	-	-	-	-	-	-
**Contact with ape feces**	70.8%	29.2%	-	-	-	-	-	-
**Wild apes around household**	22.9%	77.1%	-	-	-	-	-	-
**Wearing shoes in the forest**	39.6%	60.4%	-	-	-	-	-	-
**Eating from the ground**	97.9%	2.1%	-	-	-	-	-	-
**Washing crops before eating**	8.3%	91.7%	-	-	-	-	-	-
**Washing hands before eating**	37.5%	62.5%	-	-	-	-	-	-
**Drinking from water sources**	-	-	-	-	-	89.6%	2.1%	8.3%
**Anthelmintic treatment**	29.2%	70.8%	-	-	-	-	-	-
**Taking plant-based drugs**	4.2%	91.7%	-	-	-	-	-	4.1%

### DNA isolation, library preparation, sequencing

First, we took approximately 0.25 g of fecal sample preserved in ethanol and evaporated the ethanol overnight at 37°C. We extracted total genomic DNA from dry fecal samples using PowerSoil DNA isolation kit (MO BIO Laboratories, Qiagen company, USA) and amplified ribosomal DNA (rDNA), specifically the variable section of rDNA (internal transcribed spacer 2; ITS-2). We prepared sequencing libraries according to the protocol of Pafčo et al. [16], using two-step PCR following the Fluidigm Access Array primer design. We processed each sample in duplicate and included two negative and three positive controls according to the protocol. We sequenced the final libraries using the Illumina MiSeq platform (Illumina MiSeq Reagent Kit v2, sequencing 500 cycles of 2 x 250 bp paired-end reads). Additionally, we created a large metadata table containing sample identification (ID), collection site and host species.

### Data processing and statistics

We trimmed raw.fastq sequences using Skewer [42] and followed by paired-end reads assembly in PEAR merger [43]. We eliminated low quality sequences (with expected error rate > 1%) from the dataset. We detected ITS-2 amplicon sequencing variants (ASVs) and estimated sample relative abundances using software dada2 [44]. Using dada2’s algorithm, sequences inconsistently present in both duplicates were marked as potential artifacts (e.g., sequences with low template content, chimeras or sequencing errors) and removed from downstream analyses (5–7% of sequences after quality control). We searched for corresponding sequences via standalone BlastN (performed on the NCBI nt database, which was downloaded on 10^th^ February 2020); we excluded environmental or uncultured samples from the database and filtered out all blast hits with < 85% identity and < 90% coverage from the file. We downloaded taxonomy for blast hits using taxize package [45], and used the created reference database to assign a taxonomic classification in our dataset via dada2’s AssignTaxonomy method, implementing a Naïve Bayesian Classifier algorithm [46].

We merged the resulting taxonomy table with our metadata table in RStudio (https://www.rstudio.com); into a single phyloseq object, suitable for downstream analyses. We executed all data analyses in the statistical software RStudio. We de-noised the raw dataset (variants unclassified up to “family” level and “non-strongylid” were removed from the dataset) and used a generalized linear model (GLM) with quasipoisson error distribution to test differences in alpha diversity, evaluated as number of ASVs per sample, among the studied hosts. Additionally, we employed post-hoc testing (Tukey) to identify levels of factorial response that differ from each other. Moreover, we measured the alpha diversity by Shannon’s and Simpson’s indexes; we defined community composition as prevalence and relative representation of ITS-2 ASVs using Jaccard and Bray-Curtis ecological distances. In order to prevent negative eigenvalues during computation, we performed square root transformation of the dataset. We then performed Principal coordinate analysis (PCoA) on both Jaccard and Bray-Curtis dissimilarities. To test the interspecific differences in strongylid nematode community compositions among the hosts, we executed permutational analysis of variance (PERMANOVA), followed by analysis of similarity (ANOSIM). We implemented Multivariate general linear models (GLMs) from the R package mvabund [47] to search for community-wide divergence and identification of significant ASVs that varied due to the different host species effect. For better resolution, we constructed a diagram showing proportion of reads for significant variants. We further implemented GLM testing with quasipoisson error distribution, followed by PERMANOVA and ANOSIM to evaluate the impact of all factors from the questionnaires (Table 1) on the strongylid alpha and beta diversity in humans.

## Results

### Overall characteristics of the dataset

We analyzed fecal samples of humans (*n =* 48), western lowland gorillas (*n =* 60) and central chimpanzees (*n* = 31). In total, 2,943,087 high-quality reads were identified, with a median sequencing depth per sample of 15,612 (minimum = 9, maximum = 375,905). After duplication of the obtained sequencing data from negative control samples, no ITS-2 strongylid reads were found. Taxonomic assignment revealed 65 ITS-2 amplicon sequencing variants (ASVs), including at least five strongylid genera (Table 2). Thirty-two unassigned variants (present in 45% of samples) were tentatively classified as being closest to *Nematodirus* sp. or *Travassostrongylus* sp.; however, the sequence identity and match scores were low (84.1% and 76.8%, respectively), thus those variants probably do not represent these genera and could possibly indicate novel nematode species in the sample.

**Table 2:** List of identified strongylid nematodes found in studied hosts, sequences NCBI Accession numbers and reference.

Family	Genus	Species	NCBI Accession	Reference
**Chabertiidae**	*Oesophagostomum*	*Oesophagostomum stephanostomum* type I	KR149648.1	Cibot et al. [12]
	*Oesophagostomum*	*Oesophagostomum stephanostomum* type II	AB821022.1	Makouloutou et al. [62]
	*Oesophagostomum*	*Oesophagostomum* sp.	KR149658.1	Cibot et al. [12]
	*Ternidens*	*Ternidens deminutus*	AJ888729.1	Schindler et al. [64]
**Ancylostomatidae**	*Necator*	*Necator americanus*	LC088287.1, LC036563.1 MG256601.1	Hasegawa et al. [34]; Hasegawa *unpubl*.; Jariyapong & Punsawad *unpubl*.
	*Necator*	*Necator gorillae*	LC088299.1	Hasegawa et al. [34]
	*Necator*	*Necator* sp.	AB793535.1	Hasegawa et al. [33]
	*Ancylostoma*	*Ancylostoma* sp.^†^	LC036567.1	Hasegawa *unpubl*.
**Trichostrongylidae**	*Trichostrongylus*	*Trichostrongylus* sp. type I	Unassigned^‡^	NA
	*Trichostrongylus*	*Trichostrongylus* sp. type II	LC185220.1	McLennan et al.[66]
**Unclassified**	Unclassified	Unclassified	Unassigned^§^	NA

^†^Closest hit *A*. *ceylanicum* (similarity 95.5%)

^‡^Closest hits *T*. *vitrinius* (similarity 98.48%), and *T*. *colubriformis* (similarity 97.34%)

^§^Probably two taxa: closest hits *Nematodirus* sp. (similarity 84.1%) and *Travassostrongylus* sp. (similarity 76.8%)

### Composition of strongylid communities

**Fig 1:**
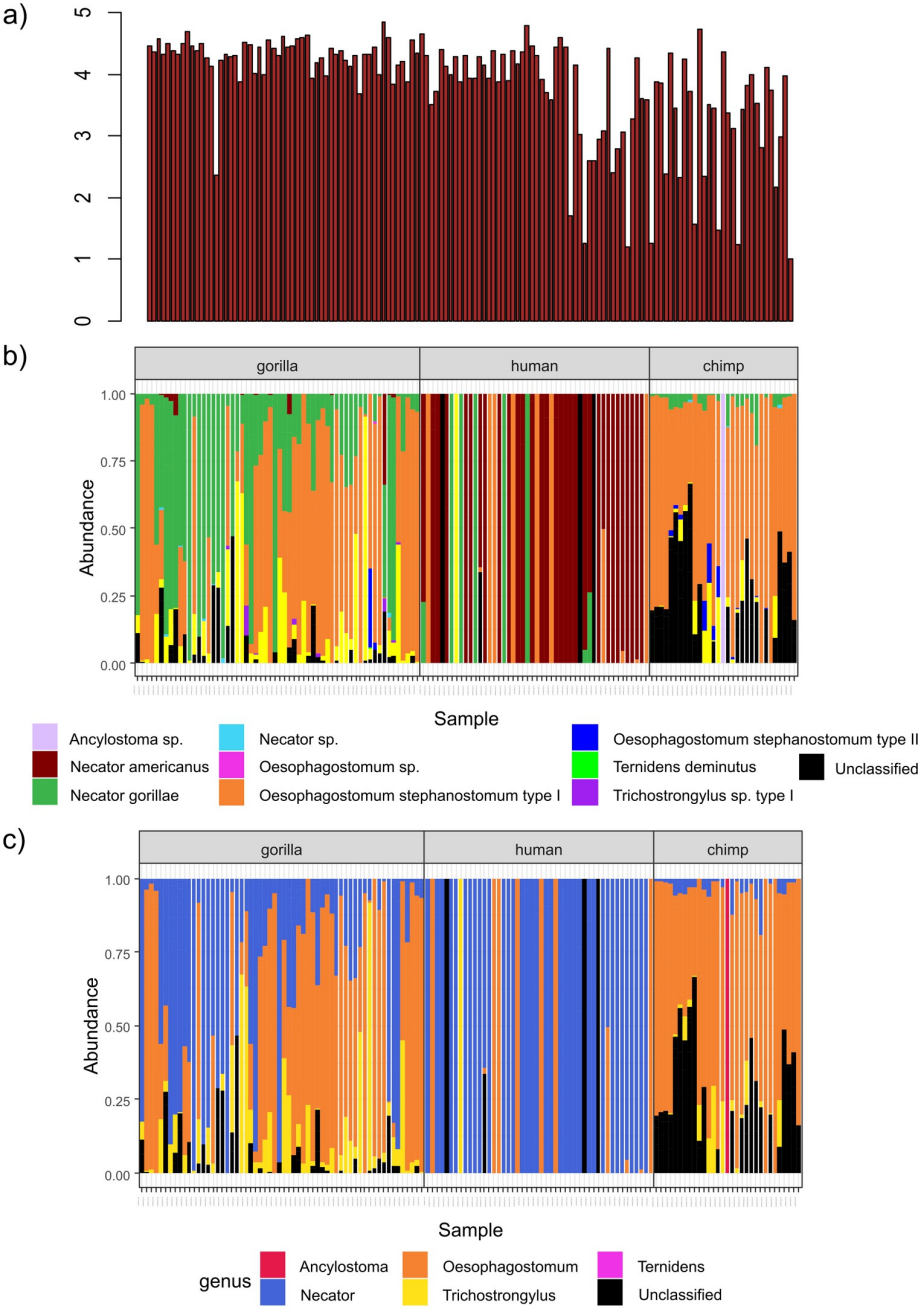
Bar plots showing a) number of reads in each individual sample on a log10 scale b) relative community composition of strongylid nematodes in examined samples at the species level, c) relative community composition of strongylid nematodes in examined samples at the genus level. Each column represents a sample. Numbers of reads (a) / relative abundances (b, c) of reads are depicted as color panels.

The most prevalent variants belonged to three genera: *Necator*, *Oesophagostomum* and *Trichostrongylus* (Table 3). A bar graph visualizing relative abundances of strongylid variants for all studied individuals revealed interspecific differences in the composition of strongylid nematode communities according to host species (Fig 1). Humans were predominantly infected by *N*. *americanus* (66.7%; median relative abundance of reads x = 3,340, min. = 143, max. = 375,811), while *N*. *gorillae* variants were less common (16.7%; x = 361, min. = 116, max. = 18,068). A significant portion of human infections also included *O*. *stephanostomum* type I (27.1%; x = 35, min. = 9, max. = 3,809). *Trichostrongylus* sp. type II (2.1%; 870 reads) and four unassigned variants (8.3%; x = 22, min. = 16, max. = 207) were also found in humans. Strongylids in great apes were dominated by variants of *N*. *gorillae* (overall prevalence 91.9%; x = 1,872, min. = 47, max. = 110,743), *Oesophagostomum stephanostomum* type I (89.3%; x = 13,209, min. = 15, max. = 49,181), *Trichostrongylus* sp. type II (69.0%; x = 863, min. = 16, max. = 38,774) and unassigned variants (67.9%; x = 3420, min. = 63, max. = 32,817). *Necator americanus* variants were found only in gorillas (31.7%; x = 107, min. = 29, max. = 7,646), while there was no evidence for *N*. *americanus* in chimpanzees. Additionally, unidentified variants of *Necator* species (neither *N*. *americanus* nor *N*. *gorillae*) were detected in great apes (13.3% in gorillas; 9.7% in chimpanzees; x = 130, min. = 50, max. = 252). Three taxa were recorded in low prevalence and with reads only evident in gorillas (*Oesophagostomum* sp. 400 reads; *Trichostrongylus* type I x = 688, min. = 52, max. = 1,832; and *Ternidens deminutus* 45 reads) and one taxon was detected only in a chimpanzee (*Ancylostoma* sp. 228 reads). We found eight ASVs shared between humans and great apes (8.25% of all observed ASVs), suggesting zoonotic transmission: two *N*. *gorillae* variants, one *O*. *stephanostomum* type I variant, and one *Trichostrongylus* sp. type II variant were found in humans, gorilla and chimpanzees, while four *N*. *americanus* variants were shared only between humans and gorillas.

**Table 3:** List of numbers of identified amplicone sequence variants (ASVs), their proportion of total reads, numbers of infected hosts and ASV prevalence among host species.

Parasite taxa	Number of identified ASVs	Total reads proportion (%)	Number of ASVs in humans	Number of ASVs in gorillas	Number of ASVs in chimpanzees	Prevalence in humans (%)	Prevalence in gorillas (%)	Prevalence in chimpanzees (%)
**Oesophagostomum stephanostomum** **type I**	16	41.1	1	12	11	27.1	81.7	96.8
**Oesophagostomum stephanostomum** **type II**	3	0.7	0	2	3	0	6.5	35.5
**Oesophagostomum sp.**	1	> 0.1	0	1	0	0	1.7	0
**Necator americanus**	16	21.7	15	5	0	66.7	31.7	0
**Necator gorillae**	14	20.0	2	14	4	16.7	96.7	87.1
**Necator sp.**	8	0.1	0	6	3	0	13.3	9.7
**Trichostrongylus sp. type I**	3	0.2	0	3	0	0	13.3	0
**Trichostrongylus sp. type II**	2	6.3	1	2	1	2.1	76.7	61.3
**Ancylostoma sp.**	1	> 0.1	0	0	1	0	0	3.2
**Ternidens deminutus**	1	> 0.1	0	1	0	0	1.7	0
**Unclassified**	32	10.0	4	18	17	8.3	58.3	77.4

### Alpha and beta diversity

**Fig 2:**
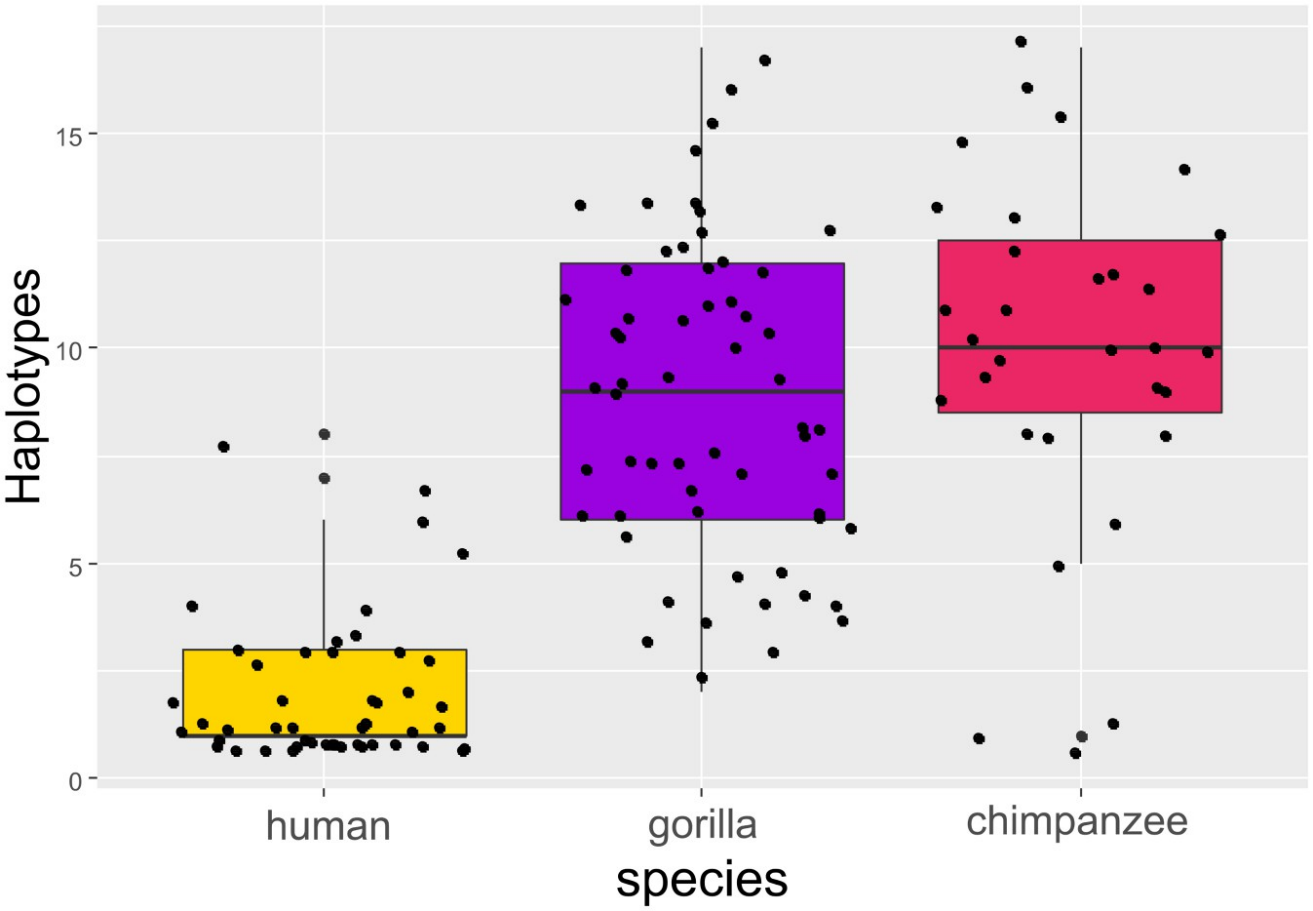
Alpha diversity of strongylid nematode communities, boxplot of amplicone sequencing variants (ASVs) counts for each sample (dots) according to host species. Different letters above boxes indicate statistically significant differences according to GLM test.

**Fig 3:**
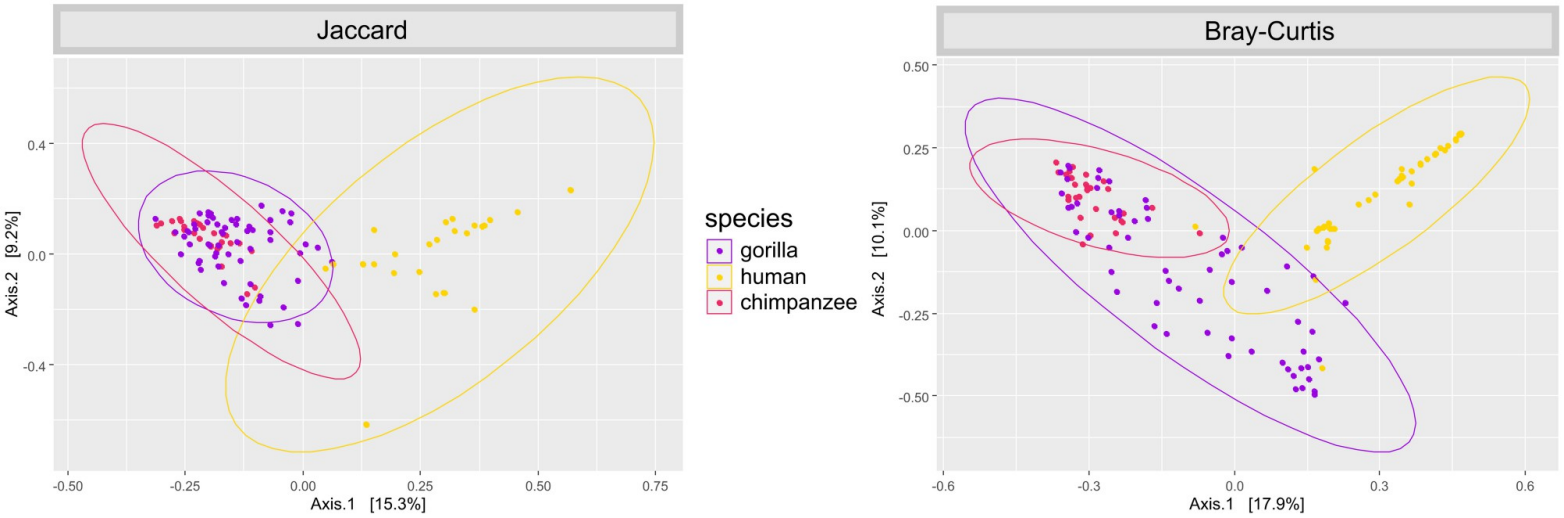
PCoA ordination diagrams of beta diversity of strongylid nematode communities based on Jaccard ecological distance: Presence/absence of amplicone sequencing variants (ASVs); Bray-Curtis ecological distance (relative abundances of reads).

**Fig 4:**
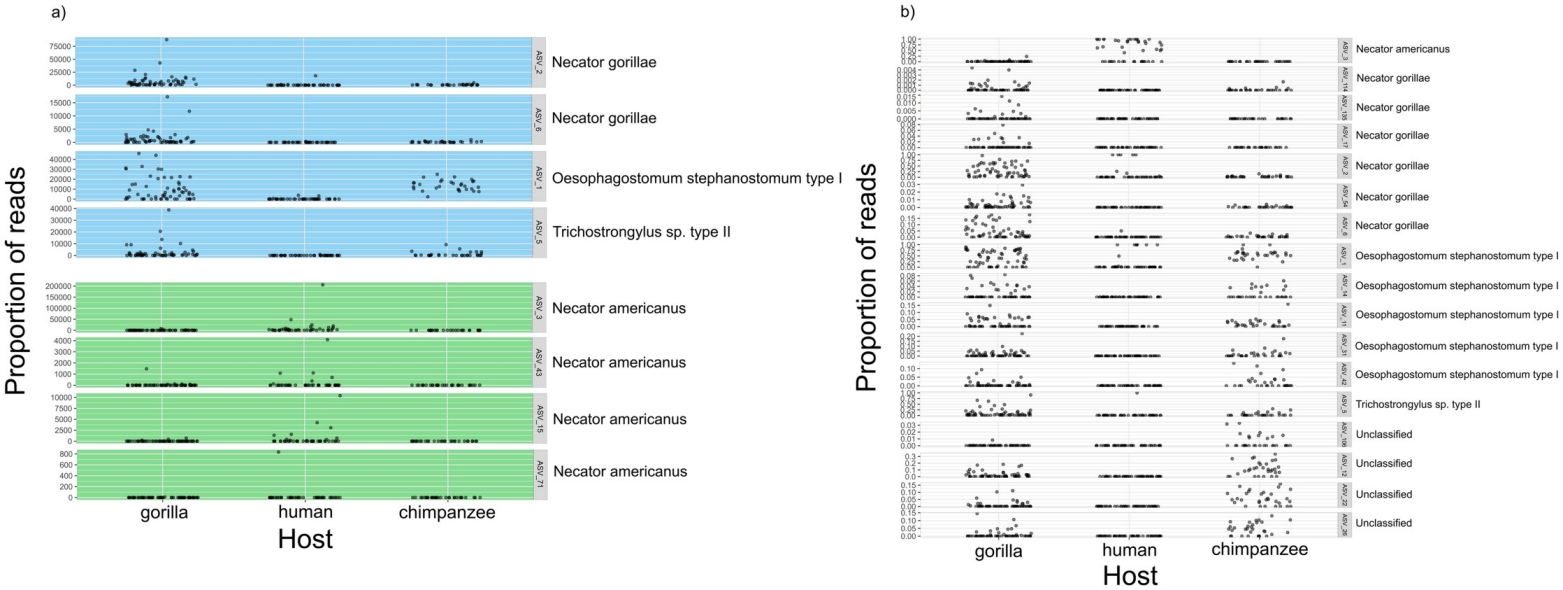
Plots showing relative abundance of ITS-2 amplicone sequencing variants (ASVs) indicated by a) shared ASVs between humans and great apes; ASVs shared between all three studied groups are highlighted in blue; ASVs shared between humans and gorillas are highlighted in green, b) Mvabund analyses as a driving force of differences among studied hosts.

Variant diversity (*x*′ = 7; min. = 1, max. = 17) differed among the studied hosts (GLM: F_(2,138)_ = 203.36, p < 0.0001). Variant diversity in humans was lower compared to both species of great apes (Tukey post-hoc testing: p = 0.0001 for all pairwise comparisons) (Fig 2), while there was no evidence of significant differences between gorillas and chimpanzees (p > 0.3). PCoA diagrams based both on Jaccard and Bray-Curtis ecological distances confirmed clear differences between humans and great apes in both composition and relative abundance of strongylid ASVs (Fig 3). Significant differences between different host species in the composition of their strongylid nematode communities were further confirmed by PERMANOVA (Jaccard: F_(2,138)_ = 11.655, p = 0.001; Bray-Curtis: F_(2,138)_ = 14.644, p = 0.001) and ANOSIM (Jaccard: R = 0.4456, p = 0.001; Bray-Curtis: R = 0.4204, p = 0.001) tests. Tukey post-hoc testing revealed significant differences between humans and other great apes for both Jaccard and Bray-Curtis (p < 0.01 for all pair-wise combinations) distances. Within great apes, there was no statistically significant result for Jaccard (p = 0.36) indicating roughly the same composition of strongylid ASVs; however, results for Bray-Curtis indicated differences in relative abundances (proportion) of ASVs between great apes (p < 0.001). Mvabund testing confirmed the interspecific differences (mvabund: ΔDF = 2, χ2 = 1002.371, p = 0.001) and identified 17 ITS-2 ASVs with whose different relative abundances were the main driving force of diversity between different host species in contrast to shared haplotypes (Fig 4a and 4b). Differences among hosts were mainly due to greater frequencies of *O*. *stephanostomum*, *N*. *gorillae*, *Trichostrongylus* type II and unclassified strongylids in great apes, whereas *N*. *americanus* was most frequent in humans.

We found no significant impact of behavioral or hygiene habits of the local people on either strongylid alpha or strongylid beta diversity (GLM: p > 0.05; PERMANOVA: p > 0.05; ANOSIM: p > 0.05).

## Discussion

### Strongylid community composition

We explored strongylid diversity and transmission patterns in humans and great apes sharing the same habitat in an unprotected area at the northern border of the Dja Faunal Reserve (Dja FR), Cameroon. Using the ITS-2 locus for identification, general taxonomic assignment revealed 95 strongylid ITS-2 amplicon sequence variants (ASVs), of which we could classify 65 at the genus/species level. We are aware of the limitations of the ITS-2 marker [17–19] and attempted to eliminate errors by running all samples in duplicates, excluding inconsistently present sequences from the dataset and using negative controls. We did not set a threshold for the number of reads per sample to detect rare strongylid taxa. We know that the threshold in this case is somewhat controversial (due to the low number of reads in some samples/ASVs). However, no official threshold has been set yet and different thresholds and settings are used in other studies [22,48].

In contrast to our previous study in Dzanga Sangha Protected Areas (DSPA), Central African Republic, where only two variants (from the total of 85) remained unassigned [4], our data from Dja FR contained 32 unassigned ASVs on the genus level. This suggests a more diverse strongylid fauna in Dja apes and humans, and further indicates that strongylid nematodes are a rather understudied group with unexplored diversity.

Overall, the composition of strongylid communities found in Dja remained generally consistent with previous studies, suggesting that *Necator* and *Oesophagostomum* are the most prevalent strongylid genera in African apes and humans [4,12,16,24,32,34,49,50], but unlike in previous studies, these were followed by *Trichostrongylus* and unassigned genera. Dja apes were mostly infected by variants of *O*. *stephanostomum* and *N*. *gorillae*, both commonly found in great apes [4,12,24,32,50]. Humans were mostly infected by *N*. *americanus* variants, confirming that *N*. *americanus* is the dominant human-specific hookworm in general [51]. Great apes exhibited higher strongylid diversity than humans and mixed infections of more than one strongylid species were frequently observed, which is consistent with previous findings in DSPA [4]. On the contrary, Vlčková et al. [52] used the same sample set as the present study and observed a higher alpha diversity of *Entamoeba* (protozoan parasite) communities in people living in the Dja compared to co-occurring great apes.

Mason et al. [24] employed HTS techniques to survey strongylid nematodes of wild western lowland gorillas in five distinct localities across the Congo Basin. The authors [24] observed lower strongylid diversity in western lowland gorillas in Dja compared to other study areas (including DSPA, CAR), and explained this to be due to the impact of greater anthropogenic disturbance on strongylid communities in the unprotected periphery of Dja FR compared to protected sites. Traditionally, parasites are thought to have negative effects on the host; however, they are a natural part of the host environment due to millions of years of evolution [53] and it has more recently been speculated that the loss of parasitic symbionts in industrialized human populations may contribute to an increase in autoimmune diseases [54]. It appears that a loss of parasitic symbionts occurs in areas of increased anthropogenic pressure [55]; however, studies employing better characterization of the anthropogenic disturbance across sites are warranted.

### Necator

We discovered six ASVs of *Necator* spp. (6.19% from the total of 97 ASVs found) being shared between multiple hosts. Besides the human hookworm *N*. *americanus*, other *Necator* species (*N*. *exilidens*, *N*. *congolensis* and *N*. *gorillae*) have been reported in great apes [56–58] and also in humans in Africa [4,33,59]. Four variants of *N*. *americanus* and two variants of *N*. *gorillae* were found co-infecting humans and great apes, suggesting ongoing transmission events previously described in the tropical forest ecosystem in DSPA and Moukalaba-Doudou National Park [4,16,33,34]. While *N*. *americanus* variants were found mostly in humans, four of them were shared with gorillas; this demonstrates that *N*. *americanus* is not a solely human-specific parasite. Moreover, such a finding was previously observed in African great apes [4,24,33,34]. *Necator gorillae* variants were found predominantly in great apes, suggesting its probable ape origin, but they were also shared with humans. The *N*. *gorillae* variants corresponded to those previously found in gorillas in Gabon [34]. We did not find evidence of *N*. *americanus* infecting wild chimpanzees, which supports a previous hypothesis of a lower susceptibility of chimpanzees to *N*. *americanus* infections [34], despite some cases of chimpanzee infections having been previously recorded [4,60]. Additionally, we found a few variants of undetermined *Necator* sp. in Dja apes corresponding to variant III-1 first found in humans in DSPA by Hasegawa et al. [33], later reported in western lowland gorillas across several African localities [24,34]. Hasegawa et al. [33] speculated that variant III-1 sequences may represent *N*. *congolensis* or *N*. *exilidens*, previously described in chimpanzees [56,57]; however, the original descriptions of *N*. *congolensis* or *N*. *exilidens* were made at the beginning of the last century, and even “traditional” morphology-based taxonomy of *Necator* non-*americanus* species remain unclear [59]. Several *Necator* species are clearly capable of infecting both humans and NHPs, at least in habitats where they share the same environment. However, the exact species diversity is not known, nor is the epidemiology and ability (particularly of the non-*americanus* species) to spread in human populations. Therefore, large-scale studies covering multiple populations of wild great apes, other NHPs and humans, with utilization of advanced HTS tools combined with modern morphological characterizations will be required for better understanding of *Necator* epidemiology.

### Oesophagostomum

Two *Oesophagostomum* species are commonly found in great apes and humans throughout Africa–*O*. *stephanostomum* [12,50] in great apes and *O*. *bifurcum* in humans, especially in West Africa [61]; other *Oesophagostomum* species have been recorded, but they are much rarer [11,13]. We recorded one variant of *O*. *stephanostomum* type I shared among great apes and humans in Dja, corresponding to the variant infecting NHPs and humans in Kibale, Uganda [12]. This means our finding is the second observation of *O*. *stephanostomum* in humans, providing evidence that *Oesophagostomum* species have zoonotic potential under suitable circumstances. Pafčo et al. [4] also reported *O*. *stephanostomum* infecting NHPs in DSPA but not in humans, thus suggesting its ape origin. This is also supported by Mason et al. [24], who found *O*. *stephanostomum* in high prevalence in western lowland gorillas across several African localities. We only found the second *Oesophagostomum* group (*Oesophagostomum stephanostomum* type II) in great apes; this group was previously described in western lowland gorillas in Moukalaba-Doudau National Park (MDNP), Gabon [62] and in Dja FR, Cameroon (using the same gorilla dataset as was used for this study [24]). We only found the other variants of undetermined *Oesophagostomum* sp. in one gorilla. They correspond to *Oesophagostomum* sequences from humans and NHPs in Kibale, Uganda [32], further recorded by Cibot et al. [12] in olive baboons in other part of Kibale, Uganda. Pafčo et al. [4], found *O*. *bifurcum* infecting mangabeys in DSPA, but not great apes; we also found no evidence of this species in great apes or humans in Dja FR, although it is known to commonly infect both humans and NHPs in Africa [24,61,63].

### Other strongylids

Other strongylid nematodes also infect humans and NHPs in Africa, such as the “false hookworm” *Ternidens deminutus*, the cyathostomine worm *Murshidia* spp. [64], strongylids belonging to Trichostrongylidae [4,65], and other pulmonary strongylids such as *Mammomonogamus* [7]. We found several ASVs of *Trichostrongylus* spp. being harbored by Dja FR great apes (their strongest BlastN matches were to trichostrongylids parasitic in sheep), and we found one variant to be shared between great apes and one human at Dja FR, corresponding to the *Trichostrongylus* variant from chimpanzees living in degraded forest fragments in Bulindi, Uganda [66]. Variants of *Trichostrongylus* were reported by Pafčo et al. [4] and Mason et al. [24] in lowland gorillas, and adult *Trichostrongylus* worms were found in necropsied mountain gorillas in Rwanda [67]. Although several cases of *Trichostrongylus* infections have been reported in humans in north-eastern Thailand, Lao People’s Democratic Republic (PDR) and urban areas of Salvador City, Brazil [68,69], human *Trichostrongylus* infections are considered rather incidental. We found one variant of *Ternidens deminutus* infecting western lowland gorillas, closely similar to the one from Mona monkeys (*Cercopithecus mona*) found in Ghana [64]; this finding also corresponded to Pafčo et al. [4], who found four *T*. *deminutus* variants infecting great apes, being closely related to the same sequence. *T*. *deminutus* is considered to be a neglected parasite of humans [64] and has also been reported in chimpanzees of Tai, Côte d’ Ivoire [5] and in western lowland gorillas of Loango National Park, Gabon and in DSPA, CAR by Mason et al. [24], thus raising questions about its origin and zoonotic potential. *Ancylostoma* duodenale is considered a human-specific parasite and was found by Pafčo et al. [4] in humans in DSPA, Central African Republic. Our data show evidence for the first chimpanzee infection by *Ancylostoma* sp. ever recorded; however, we could not specifically assign the variant to a known *Ancylostoma* species, and it was found in only one chimpanzee sample, representing 100% of total sample reads. Such homogeneity in chimpanzees is rather unusual, according to our dataset.

### Zoonotic transmission patterns

In Dja FR, humans exhibited lower strongylid alpha diversity than great apes and formed a separate cluster distinct from great apes, which was caused by dominance of *N*. *americanus* variants in both prevalence and relative abundance (measured as the proportion of sequencing reads assigned to this species). On the other hand, the strongylid communities of the two great ape species overlapped and were dominated by variants belonging to *N*. *gorillae*, *O*. *stephanostomum*, *Trichostrongylus* type II and unclassified variants. Our results corroborate those from DSPA, CAR [4], where the composition of strongylid communities was also shaped by the extent of habitat sharing, which is much more intense among species of great apes than between humans and great apes. Infective larvae (L3) of monoxenous strongylid nematodes develop in the external environment [6], thus habitat sharing increases the risk of infection and transmission between hosts. Thus, in both DSPA and Dja FR, the observed patterns of strongylid communities did not reflect the phylogenetic relationships of the hosts as they are more similar between great ape species than between phylogenetically closer humans and chimpanzees [70]. Interestingly, the composition of human and chimpanzee *Entamoeba* communities in Dja FR overlapped, while that of gorillas formed a clearly separated cluster, displaying a pattern that reflects the phylogenetic distance between the hosts [52]. Mann et al. [71] analyzed gut protists and nematodes of NHPs from various sites using the 18S phylogenetic marker. Although 18S markers cannot provide high phylogenetic resolution for strongylid nematodes [17], these results showed that gut eukaryotes (unlike symbiotic gut bacteria) were only weakly structured by primate phylogeny, similar to the case for gut mycobiome [72]. More studies are needed to understand the drivers shaping various eukaryotic gut communities of great apes and humans.

In Dja FR, we found no impact of frequency of entering the forest, interaction with great apes, clothing, hygiene, anthelmintic treatment or dietary habits; however, the implementation of questionnaires was a pilot activity, and a more detailed and rigorous social science approach would be needed to explain differences in strongylid infections among Dja FR humans. We recorded higher numbers of strongylid ASVs shared between humans and great apes in Dja FR in comparison to DSPA. This is quite surprising as the majority of human respondents in Dja FR were agriculturists while in DSPA the studied humans were contemporary BaAka hunter-gatherers and some were even employed as gorilla trackers for the Primate Habituation Programme [4,73]. Our results may therefore indicate a possible impact of rural people’s lifestyles causing anthropogenic disturbance and subsequent changes in spatial overlap between apes and humans on strongylid transmission patterns. The northern periphery of the Dja FR experiences high anthropogenic pressure as the forest is degraded and fragmented, with intense logging, hunting and farming occurring in the area [37–40]. Conversely, in DSPA, the studied apes inhabited strictly protected parts, namely the Dzanga sector within Dzanga-Ndoki National Park in DSPA, CAR [4,16,24,33,74]. The agricultural fields of the northern periphery of Dja FR attract wildlife, including apes, which can result in crop-raiding, and both humans and apes can defecate around fields [37,75]. Local people often walk barefoot through Dja agricultural fields and eat crops straight from the ground without washing them (Table 1). Together with almost no anthelmintic treatment and poor sanitation and hygiene rules, the transmission of strongylid parasites can be greatly facilitated as *Necator*, *Oesophagostomum* and *Trichostrongylus* are parasites transmitted by skin penetration or oral ingestion [6]. People living in Sub-Saharan Africa have always shared their habitat with NHPs. Our results pertain to people with agricultural and hunter-gatherer lifestyles and apes inhabiting unprotected and protected areas, and indicate that ecological, social and even political economic changes resulting in greater pressures on wildlife habitats and changes in spatial proximity between wildlife and humans have created opportunities for intensified soil-transmitted helminth spillover in both directions.

Future research should include analyses of the strongylid communities of apes from multiple areas of varying conservation status. For example, it is necessary to sample gorillas from the special reserve in DSPA, multiple-use zone with human activities surrounding the national park and Bantu people following agricultural lifestyle co-habiting DSPA, to better understand the drivers of transmission patterns in various host cohorts [76,77]. Importantly, a multi-disciplinary and anthropological–historical approach, including social science parameters, should be implemented to describe the patterns of contact and spatial overlap of humans, apes and helminths across various localities [29].

## Conclusion

We reveal complex strongylid nematode communities of great apes and humans sharing an unprotected tropical forest habitat in Cameroon. The great apes exhibited a greater diversity of the strongylid fauna harbouring more amplicon sequencing variants (ASVs) and rare variants in comparison to humans. *Oesophagostomum* and *Necator* were the dominant components of strongylid communities in all studied hosts, and the driving force of strongylid overlaps. Human communities were dominated by *Necator americanus*; although generally thought to be human-specific, this parasite was also shared by gorillas. *Necator gorillae*, originally thought to be a parasite confined to NHPs, was widespread across all studied host species, including humans. We observed a second case of *O*. *stephanostomum* infection in humans. In contrast to previous studies conducted in the DSPA, CAR, we recorded more genera and variants being shared between humans and great apes, which might be due to significant anthropogenic pressure in the periphery of the reserve, which is not protected. Most African apes occur outside protected areas [78] and thus improving the effectiveness of pathogen monitoring, conservation efforts and management not only inside, but also outside, protected areas is urgently warranted.

## Supporting information

S1 Fig Questionnaire filled by human participants about their lifestyle including frequency of entering the forest, interaction with great apes, clothing, hygiene, anthelmintic treatment and dietary habits. All participants spoke French and researchers assisted them to fill in the questionnaires.
(JPG)

S1 Table Accession numbers (from the European Nucleotide Archive) and related metadata for each sample. (XLSX)
