# Supplementary figures and images for: High diversity and sharing of strongylid nematodes in humans and great apes co-habiting an unprotected area in Cameroon
Source: PLoS Negl Trop Dis. 2023 Aug 25;17(8):e0011499. doi: 10.1371/journal.pntd.0011499 (PMC10484444; doi:10.1371/journal.pntd.0011499)

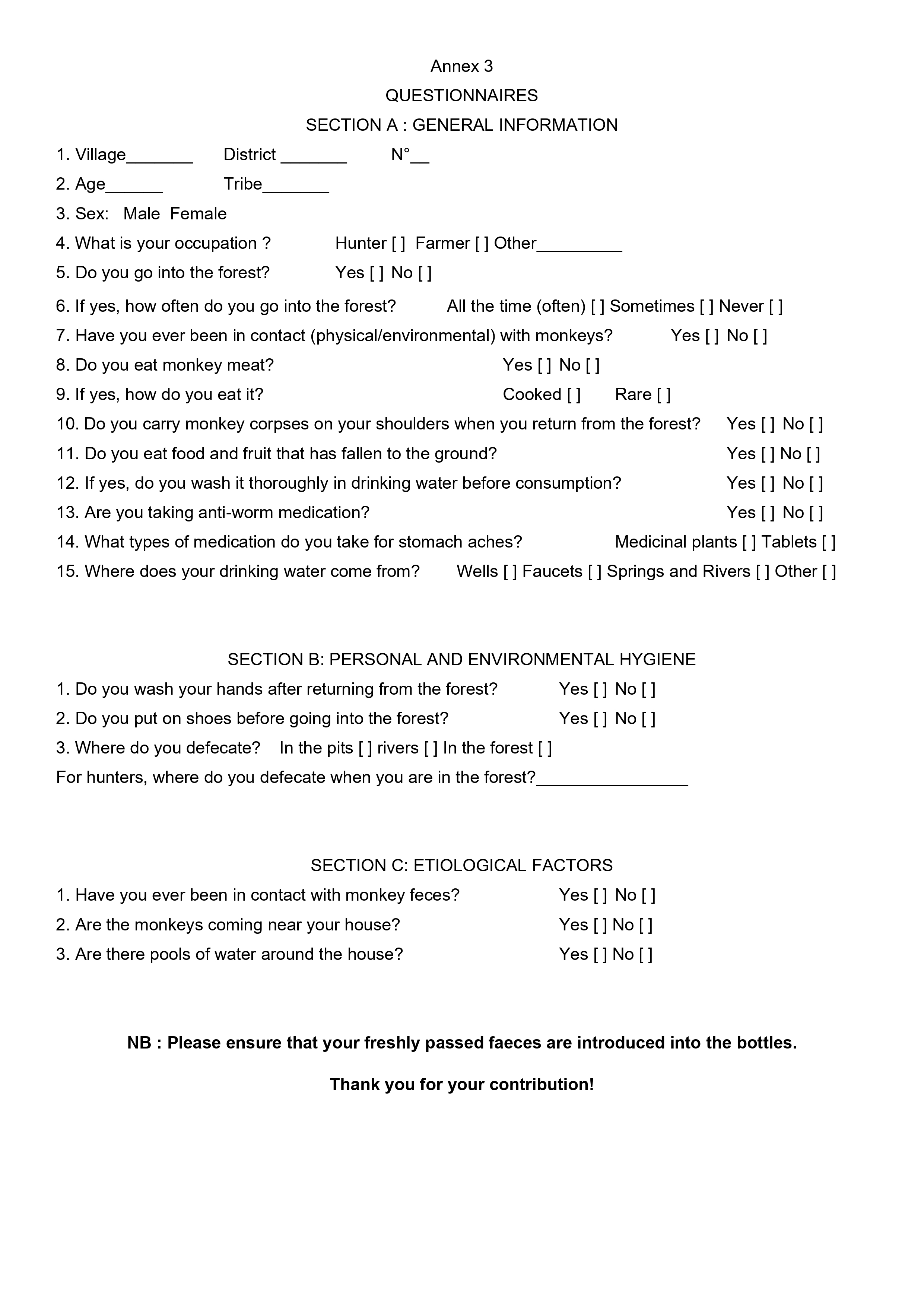

Supplement: S1 Fig — All participants spoke French and researchers assisted them to fill in the questionnaires. (JPG) [file pntd.0011499.s001.jpg]
